# Developing fluorescence sensor probe to capture activated muscle-specific calpain-3 (CAPN3) in living muscle cells

**DOI:** 10.1242/bio.048975

**Published:** 2020-09-04

**Authors:** Koichi Ojima, Shoji Hata, Fumiko Shinkai-Ouchi, Mika Oe, Susumu Muroya, Hiroyuki Sorimachi, Yasuko Ono

**Affiliations:** 1 Muscle Biology Research Unit, Division of Animal Products Research, Institute of Livestock and Grassland Science, NARO, 305-0901 Tsukuba, Japan; 2 Calpain Project, Tokyo Metropolitan Institute of Medical Science, 156-8506 Tokyo, Japan

**Keywords:** Calpain, Calpain-3, Skeletal muscle, Calpainopathy, Limb-girdle muscular dystrophy type 2A, Proteolysis

## Abstract

Calpain-3 (CAPN3) is a muscle-specific type of calpain whose protease activity is triggered by Ca^2+^. Here, we developed CAPN3 sensor probes (SPs) to detect activated-CAPN3 using a fluorescence/Förster resonance energy transfer (FRET) technique. In our SPs, partial amino acid sequence of calpastatin, endogenous CAPN inhibitor but CAPN3 substrate, is inserted between two different fluorescence proteins that cause FRET. Biochemical and spectral studies revealed that CAPN3 cleaved SPs and changed emission wavelengths of SPs. Importantly, SPs were scarcely cleaved by CAPN1 and CAPN2. Furthermore, our SP successfully captured the activation of endogenous CAPN3 in living myotubes treated with ouabain. Our SPs would become a promising tool to detect the dynamics of CAPN3 protease activity in living cells.

## Abstract (precis)

**Summary:** Our research is the first report of developing sensor probes to detect activated-CAPN3 in cultured skeletal muscle cells.

## INTRODUCTION

Calpain (CAPN) is an intracellular non-lysosomal Ca^2+^-requiring cysteine protease (EC 3.4.22.17; Clan CA, family C02), which is expressed in cells of humans and other organisms in a tissue specific or ubiquitous fashion (Goll et al., 2003). CAPNs comprise a super family and 15 different CAPN genes are found in the human genome (Campbell and Davies, 2012). CAPNs cleave a variety of substrates to modulate their structure rather than to disrupt their functions, thereby regulating intracellular activities such as cell motility (Franco and Huttenlocher, 2005; Franco et al., 2004), cell differentiation (Yajima et al., 2006) and plasma membrane dynamics (Mellgren et al., 2007; Redpath et al., 2014), etc. In contrast to other intracellular proteases such as caspases that proteolyze their substrates at relatively conservative peptide sequences in their substrates (Julien and Wells, 2017), CAPNs’ universal consensus amino acid sequences to be cleaved by CAPNs have not been identified in their substrates, i.e. CAPNs’ substrate preference motif is still ambiguous (Shinkai-Ouchi et al., 2016). Nevertheless, CAPN protease activity is strictly controlled; CAPNs cleave their specific substrate at limited site(s) to provide new function(s) to cleaved substrate (Campbell and Davies, 2012; Ono et al., 2016b). It is likely that CAPNs recognize a connecting region between structural domains rather than consensus amino acid residues in its substrate as a cleavage site (Tompa et al., 2004). As CAPN regulates cellular process through limited cleavage of substrates, CAPN is recognized as a modulator protease.

Among CAPN superfamily proteins, CAPN3 has distinct properties; CAPN3 is predominantly expressed in skeletal muscle cells and shows extremely rapid and exhaustive autolysis (Sorimachi et al., 1993). Recent studies suggest that the initial limited autolysis allows CAPN3 to assume an active conformation (McCartney et al., 2018b). While conventional CAPNs such as CAPN1 and CAPN2 are activated by Ca^2+^, CAPN3 is activated by not only Ca^2+^ but also Na^+^ (Ono et al., 2010). CAPN3 activity does not require dimerization with calpain regulatory small subunit as in the case of CAPN1 and CAPN2. Rather, homo-dimerization has been proposed as a key to become active form (Partha et al., 2014; Ravulapalli et al., 2009). In contrast to conventional CAPNs whose protease activity is blocked by the endogenous inhibitor calpastatin (CAST), CAST does not block CAPN3 protease activity but is cleaved by CAPN3 (Ono et al., 2004). Importantly, pathogenic mutations of CAPN3 cause limb girdle muscular dystrophy type 2A (Richard et al., 1995). In CAPN3 null model mice, muscular dystrophy phenotype is recapitulated (Kramerova et al., 2004; Richard et al., 2000). Furthermore, the loss of CAPN3 proteolytic activity impairs the response to muscle stress and subsequently leads to muscle degeneration (Ojima et al., 2010). Therefore, CAPN3 protease activity is essential to maintain cellular activity in skeletal muscles.

Although it is hypothesized that CAPN3 is associated with a signal transduction pathway linked with physiological stress (Ojima et al., 2010; Ono et al., 2016a) and is responsible for maintaining homeostasis in skeletal muscles (Beckmann and Spencer, 2008), the definite function(s) of CAPN3 remain(s) elusive. One of the reasons for an ambiguity regarding the real function(s) of CAPN3 is the lack of methodology to detect CAPN3 protease activity in living muscle cells. Whereas an antibody-based assay is useful to detect CAPN3 autolysis, such approach has a fundamental limitation, i.e. it does not allow monitoring of real time CAPN3 activity, particularly in living cells. Therefore, alternative approach is essential.

The Förster/fluorescence resonance energy transfer (FRET) technique has been applied for the detection of protease activity of conventional CAPNs, CAPN1 and CAPN2, to demonstrate real time proteolysis *in vitro* and *in vivo* (Bartoli et al., 2006; McCartney and Davies, 2019; McCartney et al., 2018a; Vanderklish et al., 2000). As for detection of activated CAPN3, our group has previously generated a prototype FRET-based sensor probe (SP) that is cleaved in *in vitro* test tubes by CAPN3 and CAPN3 splice variant lacking exons 6, 15 and 16 (Ono et al., 2004). In this study, we further improved FRET-based SPs to monitor protease activity of CAPN3 in living cells.

## RESULTS

### Design of SPs cleaved by CAPN3

**Fig. 1.**
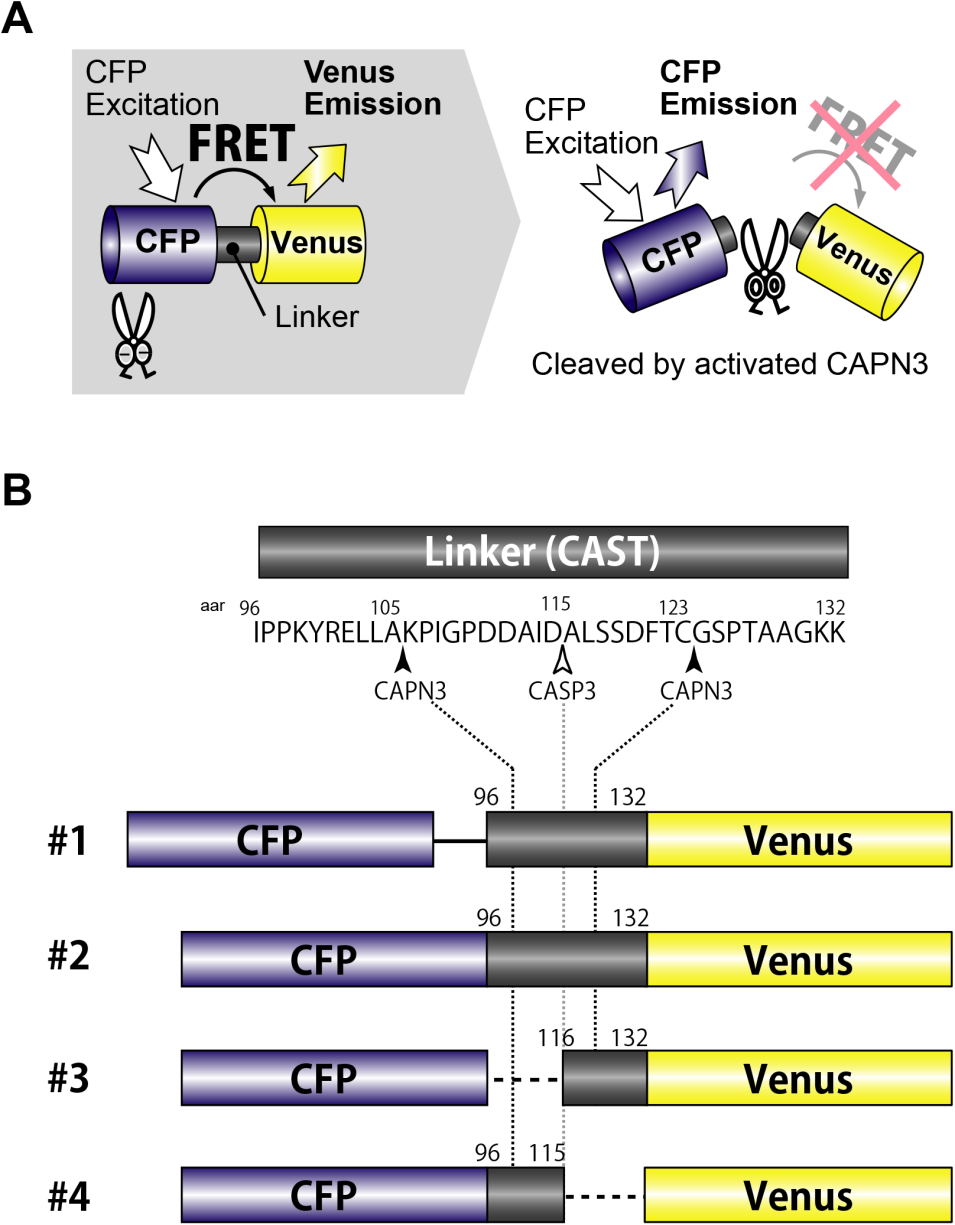
**SPs to detect activated-CAPN3.** (A) A strategy to detect activated-CAPN3 using FRET technique is represented. An SP consists of a CFP, a linker region, and a Venus. The Venus emission is increased by intramolecular FRET under the CFP optimal excitation. When the linker region is cleaved by CAPN3, intramolecular FRET is abolished, leading to a shift of emission wavelength from the Venus to the CFP. As a result, the ratio of CFP/Venus is increased. (B) In FRET-based SPs, distinct partial amino acid sequences of CAST are inserted as linker regions. Each SP (#1–#4) contains at least one CAPN3 cleavage site (black arrowheads). There is one known caspase 3 cleavage site (white arrowhead).

To capture signals of activated-CAPN3 in living cells, we developed FRET-based SPs, which consisted of enhanced cyan fluorescence protein (CFP), partial CAST sequence and mutated yellow fluorescence protein (Venus). CAST sequence was used as the linker sequence since CAST is a well-known CAPN3 substrate (Ono et al., 2004), although CAST functions as a natural inhibitor of conventional CAPNs. Four SPs with different linker sequences were designed to gain high ratio of the CFP/Venus in cells (Fig. 1).

**Fig. 2.**
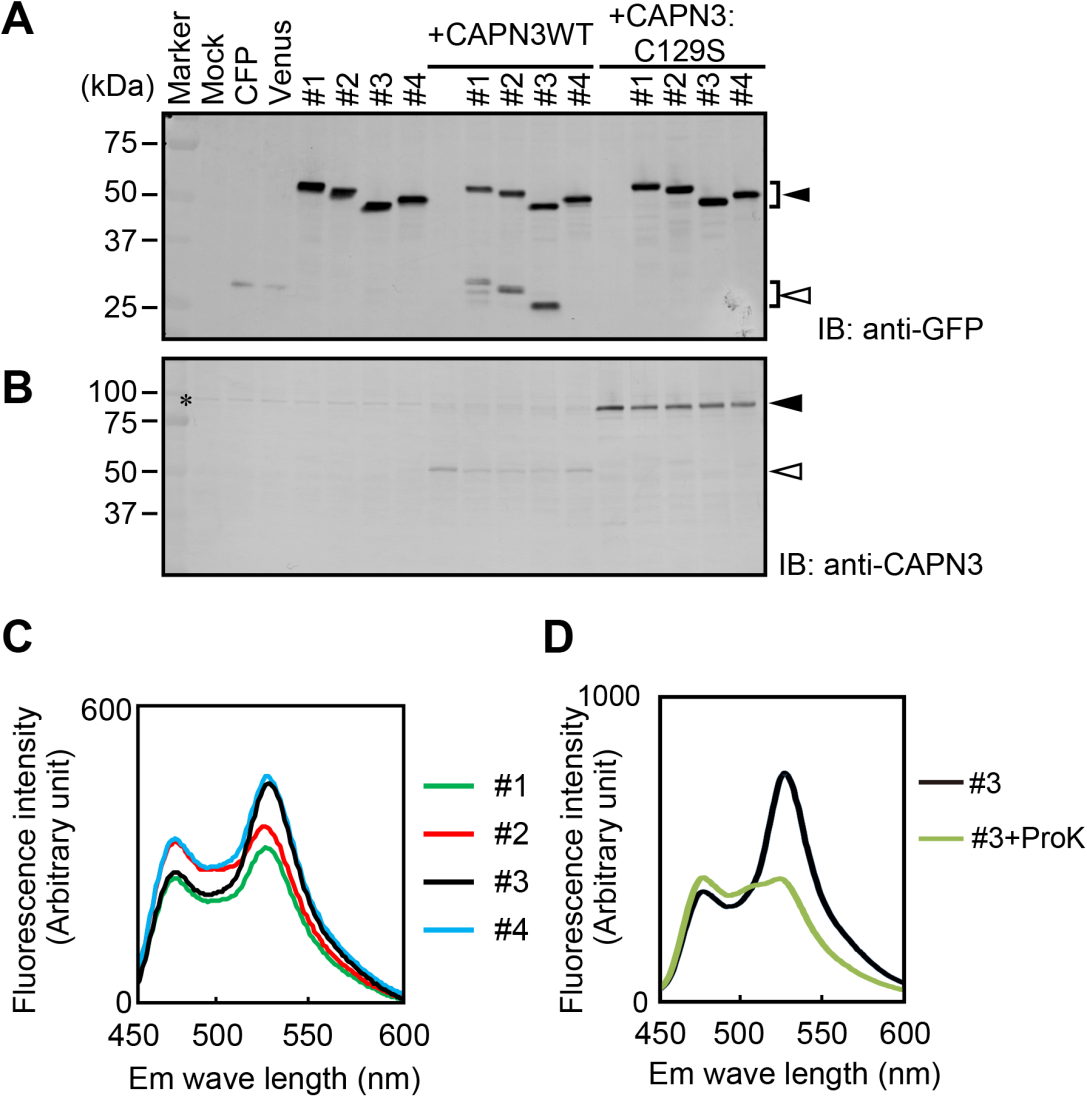
**Cleavage of FRET-based SPs by CAPN3.** (A,B) HEK293 cells were transfected with a combination of expression vectors encoding CFP, Venus, SPs (#1–#4), CAPN3WT and/or CAPN3:C129S. Immunoblot analyses revealed that intact SPs (black arrowhead in A) and cleaved SPs (white arrowhead in the blot of samples co-expressing SPs and CAPN3WT in A) were detected with an anti-GFP antibody that recognizes both CFP and Venus proteins. An anti-CAPN3 antibody captured full length CAPN3 (black arrowhead in B) and autolytic fragments of CAPN3 (white arrowhead in B). *, Non-specific bands. Note that the CFP and the Venus proteins showed slightly slow mobility compared to the SP-derived fragments since both CFP and Venus contained additional amino acid sequence before stop codon in vectors, which are absent in the SP vectors. (C) HEK293 cells were transfected with SP vectors. At 24 h post-transfection, fluorescence emission spectra from 450 to 600 nm excited by 440 nm (CFP excitation wavelength) were scanned with a fluorescence spectrometer. Emission wavelengths of the CFP (477 nm) and the Venus (528 nm) were observed in all SPs. (D) The suspension of HEK293 cells expressing SP#3 was subjected to spectral analyses. Black curve and green curve depict pre-treatment and post-treatment with ProK, respectively.

To test whether SPs were cleaved by CAPN3 in cells, expression vectors encoding SPs, wild-type CAPN3 (CAPN3WT), or protease inactive form of CAPN3 (CAPN3:C129S) were transfected into HEK293 cells. At 24 h post-transfection, cells were subjected to SDS-PAGE and immunoblotting. Ant-GFP antibody, which also recognizes CFP and Venus, detected proteolytic fragments of SPs when co-expressed with CAPN3WT in HEK293 cells. By contrast, these fragments were not observed when co-expressed with CAPN3:C129S (Fig. 2A). Anti-CAPN3 antibody captured the CAPN3 full-length bands in cells expressing CAPN3:C129S and the CAPN3 autolytic fragments in cells expressing CAPN3WT (Fig. 2B) as is reported (Sorimachi et al., 1993). These results indicate that our SPs were specifically cleaved by CAPN3WT in HEK293 cells.

### FRET properties of SPs

To evaluate our SPs in living cells, spectral properties of HEK293 cells expressing SPs were measured with a fluorometer. Maximum fluorescence intensity was observed at the Venus emission wavelength under the CFP excitation wavelength in all probes, suggesting that Venus was excited by intramolecular FRET under the CFP emission wavelength in living HEK293 cells. The SP#3 showed the highest Venus/CFP ratio at CFP excitation wavelength; the Venus/CFP ratios of SP#1, #2, #3 and #4 were approximately 1.25, 1.08, 1.72 and 1.40, respectively (Fig. 2C). Therefore, SP#3 was selected for further experiments. Next, we examined whether FRET was abolished by cleavage of the SP#3 linker region. The intensity of Venus emission was greatly reduced under the CFP excitation wavelength following proteinase K (ProK) treatment (Fig. 2D) which is previously shown to cause the loss of fluorescence intensity by hydrolyzing the linker sequence but not fluorescence proteins (Meng et al., 2008). These results demonstrated that SP#3 in fact exhibits FRET under the CFP optimal excitation wavelength.

### Little effect of CAPN1 and CAPN2 on cleavage of SP#3

**Fig. 3.**
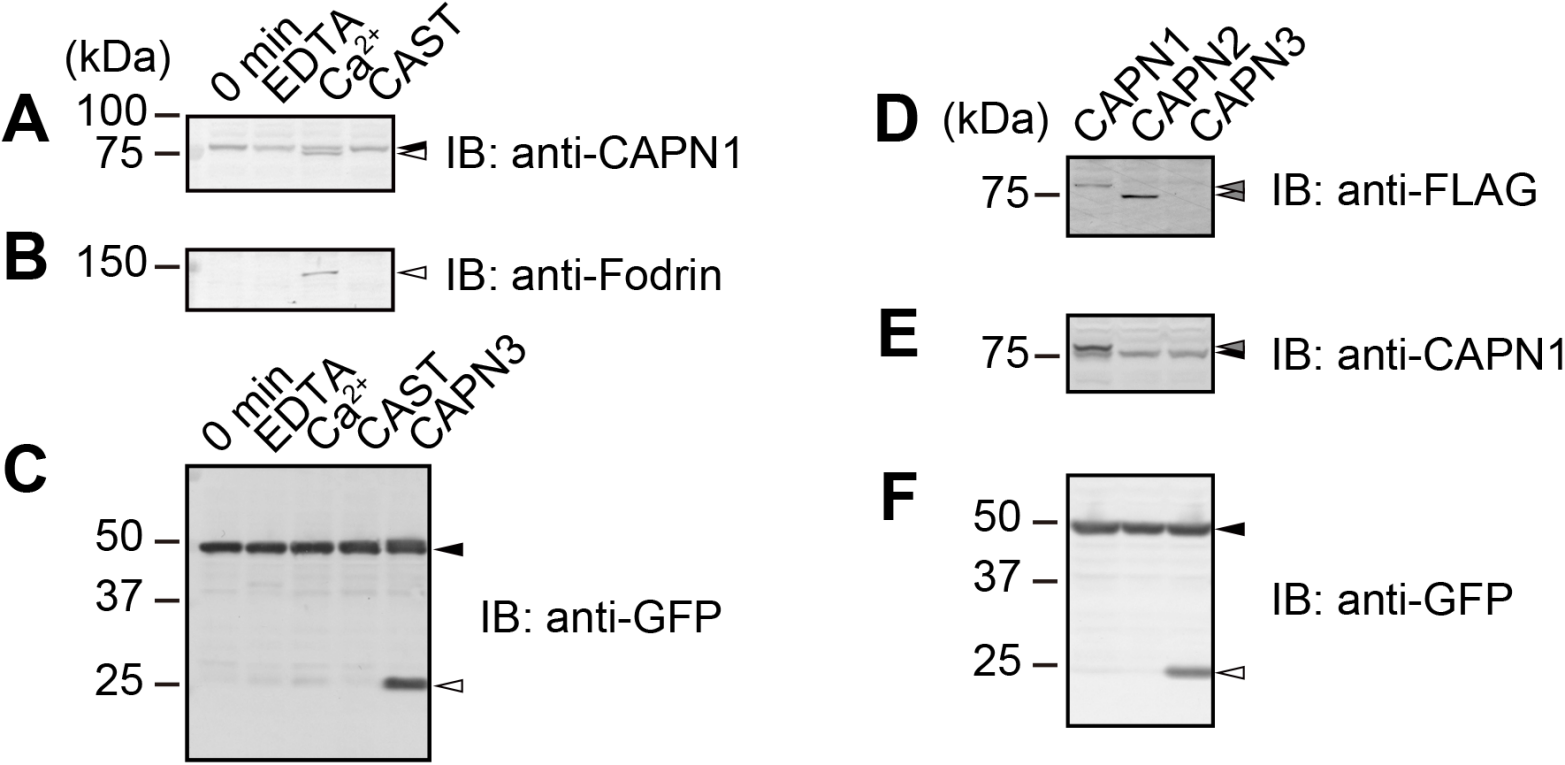
**Inefficient cleavage of SP#3 by endogenous and overexpressed CAPN1 and 2.** (A–C) Lysates of HEK293 cells expressing SP#3 were treated with 10 mM EDTA, 10 mM Ca^2+^ or 0.7 µM CAST at 30°C for 30 min to induce or inhibit CAPNs. Negative control, i.e. cell lysate without any treatment was shown as 0 min. Anti-CAPN1 antibody captured the full length (upper black arrowhead in A) and the autolytic fragments of CAPN1 (lower white arrowhead in A). Fodrin 150 kDa fragment was observed in Ca^2+^ treatment (white arrowhead in B). Anti-GFP antibody captured SP#3 signals of full length (black arrowhead in C) and 25 kDa fragment (white arrowhead in C). The lane labeled as CAPN3 is a positive control, i.e. SP3# co-expressed with wild type of CAPN3, which is also shown in Fig. 2A. (D–F) HEK293 cells were transfected with SP#3 expression vector in combination with either Flag-CAPN1, Flag-CAPN2, or Flag-CAPN3 (all wild type) expression vectors. Expression of Flag-CAPN1 and -CAPN2 were detected by anti-Flag antibody (gray arrowheads in D). Immunoblot by anti-CAPN1 antibody also confirmed that expression level of Flag-CAPN1 (gray arrowhead in E) was significantly higher than that of endogenous CAPN1 (a black arrowhead in E). Anti-GFP antibody captured SP#3 signals of full length (black arrowhead in F) and 25 kDa fragment (white arrowhead in F). Note that anti-Flag antibody did not detect full length of Flag-CAPN3 because of CAPN3 autolysis as shown in Fig. 2B.

As conventional CAPN1 and CAPN2 are ubiquitously expressed in cells, we investigated whether SP#3 was cleaved by endogenously expressed these CAPNs. Lysates of HEK293 cells expressing SP#3 were incubated with EDTA, CaCl_2_, or CAST to induce or to inhibit activation of CAPN1 and CAPN2. Antibody against CAPN1 detected two bands in the lysate with Ca^2+^ treatment (Fig. 3A). The upper band was the full length CAPN1 (a black arrowhead in Fig. 3A). The lower band was generated by autolytic ablation of the N-terminal region (a white arrowhead in Fig. 3A), indicating the activation of CAPN1. Furthermore, 150 kDa α-fodrin (spectrin) fragment, which is generated by cleavage of CAPN1 and/or CAPN2, was also observed upon Ca^2+^ treatment (Fig. 3B). In contrast to the case when SP#3 was co-expressed with CAPN3, only a trace amount of proteolysis of SP#3 was observed when endogenous CAPN1 and/or CAPN2 were activated (Ca^2+^ versus CAPN3, white arrowhead in Fig. 3C).

To examine whether SP#3 is cleaved by overexpression of CAPN1 or CAPN2, HEK293 cells were co-expressed with SP#3 and Flag-CAPN1 or -CAPN2. Overexpression of CAPNs was confirmed by anti-Flag and anti-CAPN1 antibodies (Fig. 3D,E). Interestingly, overexpression of CAPN1 or CAPN2 did not cause cleavage of SP#3 as significantly as overexpressed-CAPN3 did (a white arrowhead in Fig. 3F), i.e. SP#3 was cleaved by conventional CAPNs at almost negligible level.

### Capture of endogenous CAPN3 protease activity in living muscle cells

**Fig. 4.**
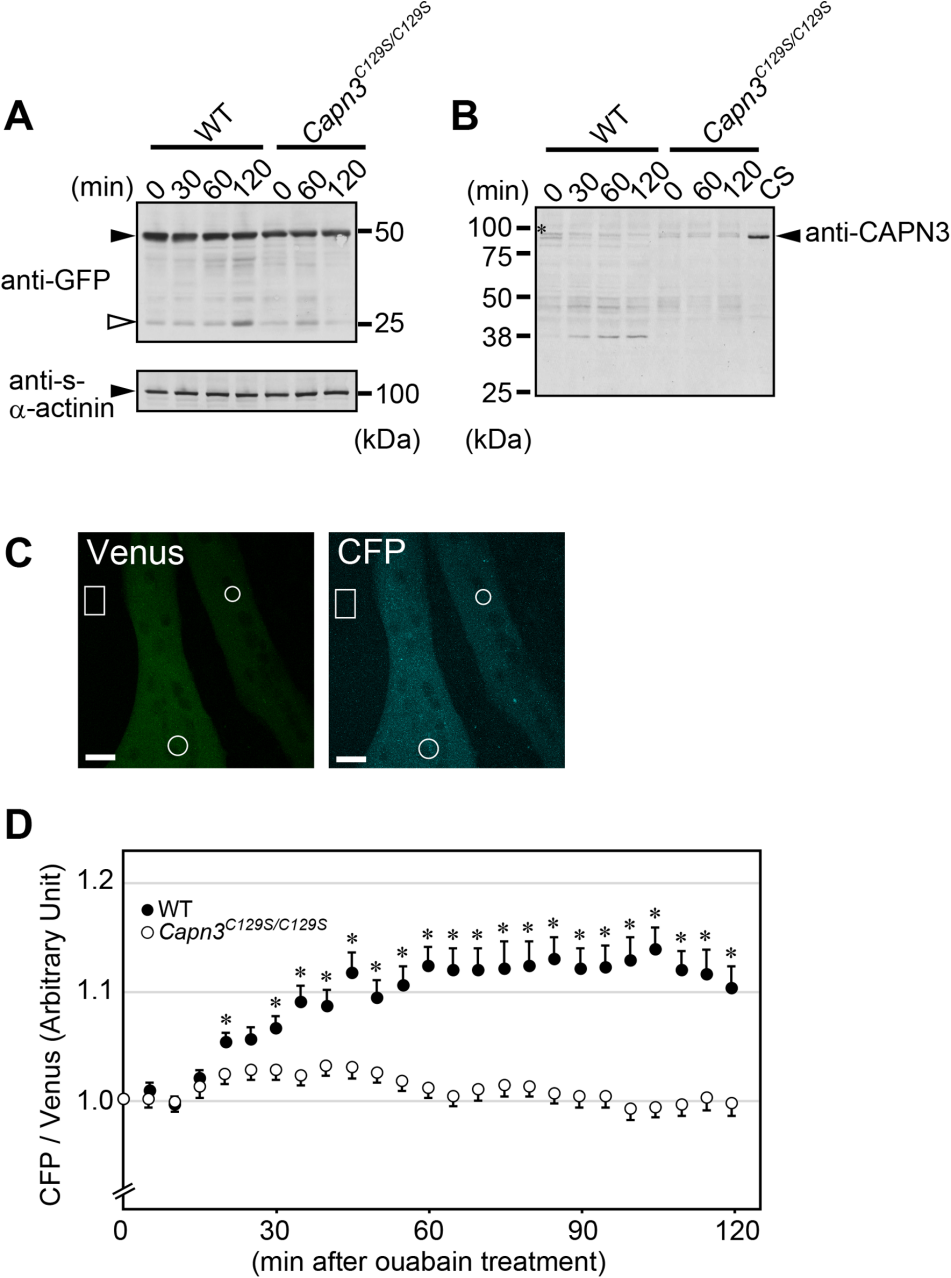
**Capturing activated-CAPN3 signals in ouabain treated-myotubes.** (A,B) Cultured skeletal muscle cells that were isolated from wild-type (WT) mice or *Capn3^C129S/C129S^* mice were transfected with SP#3 expression vector. At day 5–6, differentiated myotubes were treated with both 10 µM cycloheximide and 1 mM ouabain for the indicated time. Full-length bands of SP#3 (black arrowhead in A) and bands of their cleavage fragments (white arrowhead in A) were visualized by immunoblotting. Endogenous CAPN3 bands are indicated by black arrowhead (B). *, Non-specific bands. Sarcomeric α-actinin, one of the main Z-band components, was used as a loading control. CS depicts HEK cell lysate expressing CAPN3^C129S/C129S^, which is a protease inactive form of CAPN3. (C) Representative image of myotubes expressing SP#3 were shown. Both images of Venus and CFP were taken under the CFP excitation wavelength. ROI were shown by white circles. Background intensity was taken from the area indicated by white rectangles. Scale bars: 20 µm. (D) The ratio of CFP/Venus in each ROI of myotubes was calculated as described in the Materials and Methods section. Black and white circles indicate the data from wild-type and *Capn3^C129S/C129S^* myotubes, respectively. Ouabain was added to the medium at 0 min. * indicates significant difference (*P*<0.05) of the ratio of CFP/Venus between wild-type and *Capn3^C129S/C129S^*. Numbers of ROI, *n*=69 and *n*=61 from 40 wild-type myotubes and 36 *Capn3^C129S/C129S^* myotubes, respectively.

We tested whether SP#3 was adequately sensitive to detect endogenous CAPN3 protease activity in skeletal muscle cells. As a negative control experiment, we used cultured skeletal muscle cells that were isolated from *Capn3^C129S/C129S^* mice in which endogenous wild-type *Capn3* gene is replaced by protease inactive form of *Capn3^C129S/C129S^* (Ojima et al., 2010). Cultured-muscle cells were transfected with a vector encoding SP#3. Immunoblot studies revealed that accumulation of proteolyzed fragments of SP#3 in wild-type muscles cells upon ouabain treatment (Fig. 4A). Ouabain treatment, which increases intracellular Na^+^ concentration, caused a reduction of full length CAPN3 bands, indicating that CAPN3WT was autolyzed (Fig. 4B). On the other hand, no CAPN3 autolysis was detected in cultured *Capn3^C129S/C129S^* muscle cells (Fig. 4B). Importantly, proteolysis of SP#3 was scarcely observed in cultured *Capn3^C129S/C129S^* muscle cells, indicating that background substrate cleavage by other proteases is minimal (Fig. 4A). Although a similar trend was observed when myotubes were treated with Ca^2+^ ionophores, their extreme cell toxicity led us to choose ouabain as more preferable agent for activating CAPN3 (data not shown).

To visualize endogenous CAPN3 protease activity following ouabain treatment, myotubes expressing SP#3 were monitored by live imaging (Fig. 4C,D). In wild-type muscle cells, a rise in the ratios of CFP/Venus was captured from 30 min onward after addition of ouabain, indicating that endogenous CAPN3 cleaved SPs in myotubes. By contrast, the ratio of CFP/Venus was relatively constant in cultured *Capn3^C129S/C129S^* muscle cells (Fig. 4D). These results demonstrated that SP#3 was specifically cleaved by endogenous CAPN3 activated in cultured muscle cells that were treated with ouabain.

## DISCUSSION

CAPN3 autolysis has been used for a long time as a hallmark of activated-CAPN3. However, it is anticipated that detecting activated-CAPN3 through not CAPN3 autolysis but CAPN3's substrate cleavage enables a closer look at the function of CAPN3. Here, we showed that improved-version of FRET-based SPs successfully captured the signal of activated-CAPN3 in living cells. Although FRET-based system has been applied for real time monitoring of conventional CAPNs’ protease activity (Bartoli et al., 2006; McCartney and Davies, 2019; Stockholm et al., 2005; Vanderklish et al., 2000), this is the first report of detection of endogenous CAPN3 protease activity in cultured living muscle cells.

One of the improvements in our SPs is using Venus instead of YFP, thus the fluorescence signal is expected to be high compared to the previous construct (Nagai et al., 2002). The second advantage is the specificity of the linker in SPs that contains CAPN3's favorable amino acid residues. Although substrates of CAPN3 have not been identified in *in vivo* skeletal muscle cells, *in vitro* two CAPN3 substrates are well known, spectrin (α-fodrin) and CAST, other than CAPN3 itself (Ono et al., 2004). Partial amino acid residues of α-fodrin have been frequently used as a linker region in FRET-based SPs to monitor conventional CAPNs’ activity because CAPNs also cleave α-fodrin (Bartoli et al., 2006; Stockholm et al., 2005; Vanderklish et al., 2000). Thus, when α-fodrin sequence is used as a linker, it is difficult to determine which CAPNs, including CAPN3, cleave the FRET probes in cells. On the other hand, CAST is the endogenous specific inhibitor protein of both CAPN1 and CAPN2, but intriguingly, is a good substrate of CAPN3 (Ono et al., 2004, 2014). Therefore, to increase the substrate specificity of CAPN3, the partial amino acid sequence of CAST is a more appropriate choice for a linker of SP. Although SP#3 was cleaved by conventional CAPNs to a small (almost negligible) extent, our FRET-based SP is reasonably specific and usable for real time imaging of CAPN3 protease activity in living cells.

Our previous *in vitro* test tube assays using a prototype SP expressing COS7 cell lysates demonstrated that the ratios of CFP/YFP are approximately 1.2 with CAPN3WT (Ono et al., 2004). In the present study using SP#3, activated CAPN3 signals were detected by imaging analysis of living myotubes, however, the ratios of CFP/Venus were about 1.1, which was lower than the previous one. One of the reasons that activated-CAPN3 signals cannot be captured with high ratio of CFP/Venus is the distinct intracellular localization between CAPN3 and SPs in living myotubes. CAPN3 is mainly localized in the myofibrils (Ojima et al., 2007; Sorimachi et al., 1995) and the sarcoplasmic reticulum (Ojima et al., 2011). Approximately, only 5% of CAPN3 is present in the cytosol (Murphy et al., 2011), while exogenously expressed SPs are mainly distributed in the cytosol. Thus, the interaction between the activated CAPN3 and the SPs may occur at low frequency, leading to low detection efficiency and low CFP/Venus ratio.

Another reason might be that CAPN3 is protected from activation when it interacts with its scaffold proteins such as connectin/titin and PLEIAD in muscle cells (Ono et al., 2013, 2006). This intracellular inhibition mechanism of CAPN3 might regulate the amount of proteolytically activated CAPN3. In our previous study, however, the assays used cultured non-muscle cells for recombinant CAPN3 expression, which are devoid of such a protection system for CAPN3, i.e. equal to *in vitro* test tube assays, and activity of CAPN3 could be detected with higher ratio of CFP/YFP (Ono et al., 2004). It is also worth noting that CAPN family proteases are thought to be activated as a small fraction, in very limited regions in cells for extremely short periods (Campbell and Davies, 2012). We propose that CAPN3 also possesses this property; thus, it is not easy to capture activated CAPN3 signals in living cells.

Unfortunately, we cannot exclude the possibility that ouabain treatment leads to CAPN3 autolysis preferentially but not to efficient cleavage of SP by CAPN3. Nevertheless, even though only limited amounts of CAPN3 was activated for substrate proteolysis, the SP#3 developed by us successfully captured activated CAPN3 in living cells.

## MATERIALS AND METHODS

### Constructs

The CAPN3 SPs consisted of CFP, linker and Venus (Fig. 1A) (Nagai et al., 2002). cDNAs of partial CAST sequences and Venus were inserted into the peCFP-C1 vector (Takara Bio). SP#1, SP#2, SP#3 and SP#4 contained 96-132, 96-132, 116-132, and 96-115 amino acid residues (aar) of human CAST (UniProtKB/Swiss-Prot: P20810) as linker sequences (Fig. 1B). The SP#1 contained additional 12 aar between the CFP and the linker. Protease inactive form of CAPN3 in which the Cys residue at 129 was substituted by the Ser residue to abolish catalytic capability and was referred to as CAPN3:C129S (Ono et al., 2004). The cDNAs for full-length human wild-type CAPN1, CAPN2, CAPN3 and CAPN3:C129S were subcloned into pcDNA3.1/N-FLAG vector (Ono et al., 2004).

### Cell culture, transfection and pharmacological treatment

Experimental animals were cared for as outlined in the Guide for the Care and Use of Experimental Animals (Animal Care Committees of Institute of Livestock and Grassland Science, NARO), which the committee accepted.

HEK293 cells were cultured in Dulbecco's modified Eagle's medium (DMEM; Life Technologies) with 10% fetal bovine serum (FBS; Life Technologies) and penicillin-streptomycin.

Skeletal muscle cells were prepared from wild-type mice (C57Bl6N) and *Capn3^C129S/C129S^* mice in which the endogenous *Capn3* gene was replaced by a mutant *Capn3^C129S/C129S^* gene (Ojima et al., 2010). *Capn3^C129S/C129S^* mouse muscle cells express a proteolytically inactive CAPN3:C129S. Cells were cultured on matrigel coated-dishes in DMEM with 10% FBS for growth. After transfection, medium was changed to 5% horse serum in DMEM to induce muscle differentiation. For imaging, DMEM was switched to Phenol Red-free DMEM to avoid the intrinsic fluorescence of media.

Constructs were transfected into cells with a Lipofectamine LTX Plus reagent (Life Technologies). For live imaging of the FRET experiments, cells were treated with 10 µM cycloheximide (Sigma-Aldrich) and 1 mM ouabain (Na^+^/K^+^ ATPase inhibitor; Sigma-Aldrich). Cycloheximide was added to the medium at 30 min pre-experiment. Ouabain was added to the medium just before FRET imaging experiments to increase intracellular sodium concentration and subsequently to activate CAPN3 (Ono et al., 2010).

### Immunoblotting

Harvested cells were lysed in TED buffer [10 mM Tris/Cl (pH 7.0), 1 mM EDTA, 1 mM DTT] including protease inhibitors [2 mM PMSF (Sigma-Aldrich), 0.1 μM leupeptin (Peptide Institute), 40 μM bestatin (Sigma-Aldrich), 1.5 μM aprotinin (Sigma-Aldrich), and 0.7 μM CAST (Takara Bio)] using 27G syringe needles. For immunoblot, samples were boiled in SDS-sample buffer. For endogenous CAPN activation assay, cell lysates prepared as above were incubated with 10 mM EDTA, 10 mM CaCl_2_, 150 mM NaCl, or 0.7 µM CAST for 30 min at 30°C (Ono et al., 2010). SDS-PAGE and immunoblot analyses were performed as previously described (Ojima et al., 2007). Antibodies used in this study were listed in Table 1. Band signals were visualized using peroxidase-conjugated secondary antibodies (Nichirei Bioscience) and a POD immunostaining set (FUJI FILM Wako Pure Chemical).

**Table 1.**
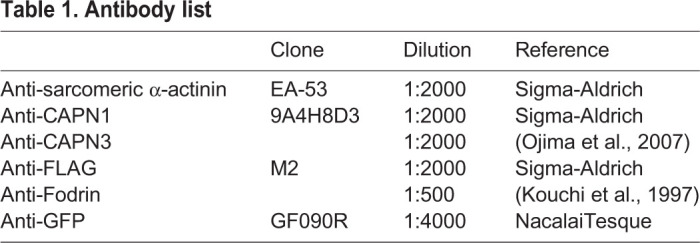
**Antibody list**

### Spectral analysis

At 24 h post transfection, HEK293 cells expressing SPs were suspended and transferred in a quartz cuvette and analyzed with a spectrophotometer RF5300PC (Shimadzu). Spectral analysis was conducted at CFP excitation wavelength 440 nm as the Venus, FRET acceptor, was excited by the CFP, FRET donor. The ratio of CFP/Venus at CFP excitation wavelength was defined as a FRET ratio. To disrupt FRET, suspended-HEK293 cells were incubated with proteinase K (ProK; NacalaiTesque) for 30 min and subsequently analyzed with a spectrophotometer since it has been shown that ProK does not degrade GFP variant proteins but degrades linker regions of SPs (Meng et al., 2008).

### Imaging and FRET analysis

At day 5–7 post-transfection with SP#3 construct, myotubes were subjected to FRET analysis by confocal laser scanning microscopy (LSM700, Zeiss). To induce CAPN3 protease activity, ouabain was added to culture media (t=0 min). During FRET analyses, cells were cultured in a microscope stage-top incubator (Tokai Hit). The x-y images were taken with 1 µm of Z interval and subsequently ten images were projected to a single image to calculate the ratio of CFP/Venus. A series of images were taken every 5 min. The SP cleavage was measured as the ratio of CFP/Venus at CFP excitation wave length in the region of interest (ROI) according to the following formula: the mean ratio=(the mean of the CFP intensity – the mean of the CFP _back ground_ intensity) / (the mean of the Venus intensity – the mean of the Venus _back ground_ intensity). Subsequently, mean ratios were normalized with the value of mean ratio at t=0 min, which is when ouabain was added to the medium.

### Statistics

Significant differences of ratios of FRET efficiency between CAPN3 and CAPN3:C129S groups were determined using Student's *t*-test. *P*-value less than 0.05 was considered statistically significant.
